# Expression Pattern of 5-HT (Serotonin) Receptors during Normal Development of the Human Spinal Cord and Ganglia and in Fetus with Cervical Spina Bifida

**DOI:** 10.3390/ijms22147320

**Published:** 2021-07-07

**Authors:** Hrvoje Punda, Snjezana Mardesic, Natalija Filipovic, Ivona Kosovic, Benjamin Benzon, Marin Ogorevc, Ivana Bocina, Kresimir Kolic, Katarina Vukojevic, Mirna Saraga-Babic

**Affiliations:** 1 Department of Diagnostic and Interventional Radiology, University Hospital in Split, 21000 Split, Croatia; hpunda@gmail.com (H.P.); kk@mefst.hr (K.K.); 2 Department of Anatomy, Histology and Embryology, School of Medicine, University of Split, 21000 Split, Croatia; smbrakus@gmail.com (S.M.); natalija.filipovic@mefst.hr (N.F.); ivona.kosovic@mefst.hr (I.K.); benjamin.benzon@mefst.hr (B.B.); marin.ogorevc2@gmail.com (M.O.); kvukojev@gmail.com (K.V.); 3 Department of Biology, Faculty of Science, University of Split, 21000 Split, Croatia; bocina@pmfst.hr

**Keywords:** spinal cord, ganglia, human development, cervical spina bifida, serotonin

## Abstract

The expression of 5-HT (serotonin) receptors (sr) was analyzed in the spinal cord and ganglia of 15 human conceptuses (5–10-weeks), and in the 9-week fetus with spina bifida. We used immunohistochemical method to detect sr-positive, apoptotic (caspase-3) and proliferating (Ki-67) cells, double immunofluorescence for co-localization with protein gene peptide (pgp) 9.5 and GFAP, as well as semiquantification and statistical measurements. Following the neurulation process, moderate (sr1 and sr2) and mild (sr3) expression characterized neuroblasts in the spinal cord and ganglia. During further development, sr1 expression gradually increased in the motoneurons, autonomic and sensory neurons, while sr2 and sr3 increased strongly in floor and roof plates. In the ganglia, sr3 expression increased during limited developmental period, while sr1 and sr2 increased throughout the investigated period. Co-expression of sr/pgp 9.5 characterized developing neurons, while sr/GFAP co-localized in the roof plate. In the spinal cord and ganglia of malformed fetus, weaker sr1 and sr2 and stronger sr3 expression accompanied morphological abnormalities. Anomalous roof plate morphology showed an excess of apoptotic and proliferating cells and increased sr3 expression. Our results indicate a human-species specific sr expression pattern, and the importance of sr1 in neuronal differentiation, and sr2 and sr3 in the control of the roof plate morphogenesis in normal and disturbed development.

## 1. Introduction

The development of the human spinal cord starts during the process of primary neurulation, when the paired neuroectodermal folds fuse to form the neural tube [1].

Following the closure of the caudal neuropore, the neural tube reaches caudally as far as the second sacral pair of somites. The remaining sacral and coccygeal somites, and the caudal parts of the spinal cord differentiate during the process of secondary neurulation from the solid cord of pluripotent cells [2] in the tail bud that subsequently cavitates to form a hollow tube [3,4,5,6]. The tail bud cells first gradually transform into the somites, the secondary neural tube, the notochord and the tail gut, and then regress by the cell death, thus enabling disappearance of the human tail [4,5,7]. Eventually, the secondary caudal neural tube fuses with the primary neural tube in a transitional zone.

During the cranial spinal cord differentiation, the three zones became distinguishable in the side walls of the spinal cord: the ventricular zone containing mitotic cells, the intermediate zone (forerunner of the grey matter) with differentiating neuroblast and glioblasts, and the marginal zone (forerunner of the white matter) containing axons which form ventral, dorsal and lateral funiculi. In the midline, the dorsal (roof) and ventral (floor) plate remain thin [1]. In contrast, the regular layers are missing in the caudal neural tube, while only the pseudostratified epithelium surrounds the small lumen [3]. Disturbances of neurulation process lead to different forms of neural tube defects, including the dysraphic spinal cord. Thus, cervical spina bifida in human embryos displays a variety of morphological changes along the cranio-caudal body axis, from the complete absence of nervous tissue in the cervical region, to the disturbed differentiation of spinal cord layers and roof plate more caudally [5]. During the neurulation process, a group of ectodermally-derived neural crest cells migrate and coalesce to form the spinal ganglia, which extend from the cervical to the sacral vertebrae [8,9], while in the region of secondary neurulation spinal ganglia are not observed [3]. However, the existing ganglion cells subsequently differentiate into the glial cells and different neuronal subtypes characterized by distinctive receptors [9,10]. In the differentiating human spinal ganglia neurons, the presence of nociceptors and mechanoreceptors has been observed already in the fifth week of human development. Ingrowth of the dorsal roots of spinal nerves into the spinal cord and formation of the efferent part of spinal nerves that reach the body periphery by the eighth week of development enables transmission of sensory information towards the CNS [9].

The morphogenesis of the human neural tube seems to be influenced by interactions between the neurotransmitters and peptides and neurons already during the early stages of development [11], including serotonin and its receptors [12]. The cell bodies of serotonin-expressing cells appear first in the brainstem and mostly project into the spinal cord. With the progression of development, the amount of serotonin increases rapidly. The function of serotonin as a growth factor is enabled by the presence of different serotonin receptors, of which the 5-HTla receptor appears only at specific times in development, indirectly acting stimulatory to serotonin neuronal growth, while 5-HT3 acts inhibitory to the development of serotonin neurons [13]. Serotonin exerts complex effects on its targets via several subtypes of 5-HT receptors, which include seven families (5-HT1 through 5-HT7) that comprise 15 receptor subtypes. With the exception of the 5-HT3 receptors, which are cation channels, all other 5-HT receptor families are members of the G protein-coupled receptor superfamily. More recent studies show that the medullary serotonergic groups of neurons project to the spinal dorsal (sensory) horn, but also to the motor and autonomic nuclei of the spinal cord and medulla. The medullary serotonergic neurons exert a major modulatory influence on nociceptive processing and motoneuron excitability, as well as to the respiratory control and autonomic functions, particularly the responses to cold [14]. In the spinal cord, serotonergic receptors are primarily located on the axon terminals. In the immature neural tube, serotonin plays a role as a growth factor by influencing both proliferation and maturation of neurons, but indirectly affecting the development of many other systems as well [13]. Serotonin might also be involved in neurite outgrowth, proliferation of glial cells and control of apoptosis [15,16,17].

Following the spinal cord injury (SCI), the degeneration of 5-HT-expressing axons caudal to the lesion site correlates with lesion severity, while rostral to the lesion 5-HT axons it displays sprouting. It seems that 5-HT has a pro-regenerative role in the injured axons and that increased 5-HT neurotransmission promotes the functional recovery after SCI [18,19,20,21,22]. Functionally, 5-HT neurotransmission in the spinal cord modulates sensory, motor and autonomic functions, but often with contradictory effects, which might be explained by activation of diverse 5-HT receptors, different brainstem origins of 5-HT projections and their terminal locations [23]. In addition, the 5-HT neurons display marked differences in their global gene expression profiles, depending on their anatomical domain and developmental lineage [24]. Thus, a loss of the 5-HT1 receptor has also been reported in human conditions such as Down’s syndrome and Alzheimer’s disease [25].

Up to now, studies using different experimental animal models have resulted in quite controversial data, which might be explained by interspecies differences, type of injury and severity of lesions analyzed. On the other hand, data on the expression pattern of serotonin receptors in developing human spinal cord and ganglia, particularly in the earliest developmental stages, are extremely scarce. Therefore, the aim of this study was to analyze the expression pattern of serotonin receptors during early stages of normal human spinal cord and ganglia development and to compare it to their expression pattern in a malformed human fetus with cervical spina bifida. The observed data indicate the human-species-specific pattern of serotonin receptor expression in developing spinal cord and ganglia and its changes in anomalies associated with disturbances of primary neurulation, such as spina bifida.

## 2. Results

### 2.1. Normal Development of the Human Spinal Cord between the 5th and 10th Developmental Week (Hematoxylin and Eosin Staining)

**Figure 1:**
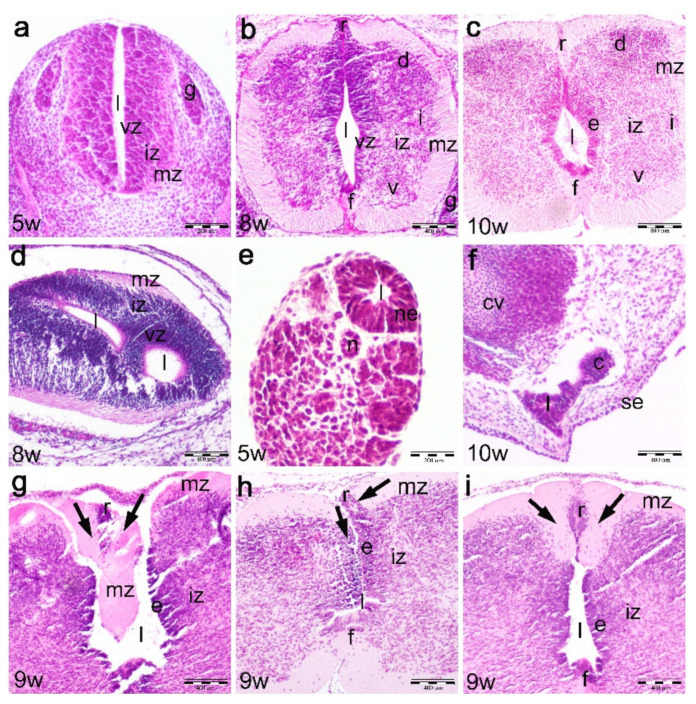
Hematoxylin and eosin staining of the normal developing human spinal cord (5th–10th developmental weeks) and a 9th week human fetus with cervical spina bifida. Developing human cranial spinal cord (sc) in the 5th–6th weeks (**a**), 7th–8th weeks (**b**) and 9th–10th weeks (**c**), transitional zone in the 8th week (**d**) and caudal spinal cord in the 5th (**e**) and 10th week (**f**): ventricular zone (vz), intermediate zone (iz), marginal zone (mz), floor plate (f), roof plate (r), lumen (l), coccygeal remnant (c), coccygeal vertebrae (cv), skin epithelium (se), dorsal ganglia (g), ventral horns (v), intermediate horns (i) and dorsal horns (d), ependymal layer (e), notochord (n). Thoracic parts of the spinal cord in the 9th week fetus with cervical spina bifida (cranio-caudal direction) (**g**–**i**): ependymal layer (e), intermediate zone (iz), marginal zone (mz), roof plate (r), floor plate (f), lumen (l). Marginal zone abnormalities (arrows) in the roof plate area (r). Magnification ×20, scale bar 200 μm (**a**,**e**); magnification ×10, scale bar 400 μm (**b**–**d**,**f**–**i**).

In the 5th developmental week, the cross section through the cranial spinal cord shows the initial signs of neuronal differentiation in the ventral part of a very thin intermediate zone, close to the very thin marginal layer. At this stage, there is still no clear distinction between the ventricular zone, containing proliferating cells, and the intermediate zone with differentiating neuroblasts. The floor plate and roof plate areas are just beginning to develop in the midline of ventral and dorsal parts of the spinal cord. The dorsal root ganglia are formed of relatively small accumulations of differentiating ganglion cells on both sides of the spinal cord (Figure 1a). During the 6th developmental week, further advancement of spinal cord differentiation is observed, with the appearance of three characteristic zones in the lateral wall (ventricular, intermediate and marginal zone), as well as roof plate and floor plate differentiation. 

In the 7th and 8th developmental week, the three zones are well developed in the lateral spinal cord walls: the ventricular zone is thinner ventrally than dorsally, indicating more advanced differentiation of ventral part of the spinal cord compared to its dorsal part, the intermediate zone, which contains neuroblasts and forms characteristic gray matter hors (ventral, intermediate and dorsal), and the marginal zone containing the axons of neurons (developing white matter). Floor plate and roof plate areas are well developed (Figure 1b) as well as the bilaterally positioned dorsal root ganglia.

In the 9th and 10th developmental week, all three layers are clearly distinguishable in the lateral spinal cord walls. The ventricular zone is thinner and now forms the ependymal layer, while the intermediate and marginal zones further widen due to advanced neuronal differentiation. The floor plate and roof plate are thickened at the expense of the marginal layer (Figure 1c), while dorsal root ganglia advance in the processes of enlargement and differentiation.

The transitional zone (joint area of cranial and caudal part of the spinal cord) in the 8th week embryo consists of the two perpendicularly oriented irregular lumens, representing the point of fusion between the two parts of the spinal cord derived by the primary and secondary neurulation (Figure 1d).

Part of the spinal cord derived during secondary neurulation appears in the tail of the 5th week embryo as an irregular lumen surrounded by neuroepithelial cells (Figure 1e). During further development, this part of the spinal cord gradually regresses and transforms into the cauda equine and filum terminale. In the 10th developmental week, the remnants of the secondary neural tube (spinal cord) can be found in the form of so-called coccygeal remnants, which are represented by an irregular discontinuous epithelial lumen between the coccygeal vertebrae and the surface skin epithelium (Figure 1f).

### 2.2. Development of the Spinal Cord in the 9th Week Dysraphic Fetus (Hematoxylin and Eosin Staining)

In the cervical area, the dysraphic part of the spinal cord is observed in the form of area cerebrovasculosa [5].

In the areas caudal to the dysraphic part of the spinal cord, the dorsal part (roof plate) of the spinal cord gradually closes along the cranio-caudal body axis. In regions closest to the cervical spina bifida, the normal morphology of the roof plate area is completely missing. Instead, the overdeveloped wedge-like part of the marginal layer penetrates into the open central canal of the spinal cord and thus closes the opening between the dorsal parts of lateral spinal cord walls. In addition, the irregularities of the three layers are observed in the lateral walls of the spinal cord (Figure 1g). The more caudally positioned section through the spinal cord reveals that the dorsal part of the spinal cord wall rolls up in order to close the roof plate area. Here, the layers in the lateral wall of the spinal cord appear asymmetric (Figure 1h). In parts of the spinal cord more distant to its dysraphic part, the roof plate is present in the form of the hypertrophic marginal layer containing a centrally positioned expansion of the ventricular zone (ependymal cells). At this vertebral level, layers in the lateral wall of the spinal cord appear more symmetrical (Figure 1i).

### 2.3. Expression of Serotonin Receptors (sr1, sr2 sr3) in the Developing Human Spinal Cord (Immunofluorescence Staining)

**Figure 2:**
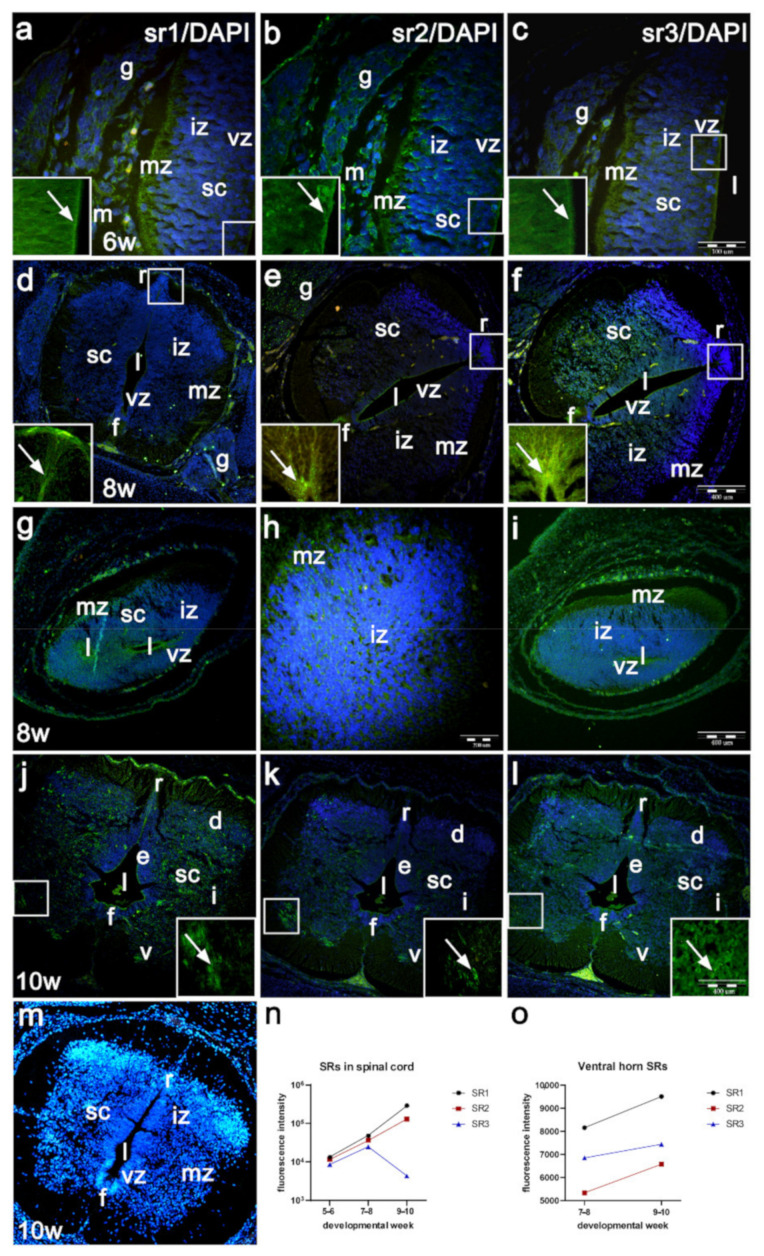
Immunofluorescence staining of developing human spinal cord with different serotonin receptors (sr1, sr2, sr3). Expression of serotonin receptors (arrows in insets), sr1 (**a**), sr2 (**b**) and sr3 (**c**) in the 5th–6th week human spinal cord (sc), in 7th–8th week cranial spinal cord (**d**–**f**), transitional zone of the 8th week spinal cord (**g**–**i**) and 10th week cranial human spinal cord (**j**–**l**) Control 10th week normal spinal cord stained only with secondary antibody and DAPI (**m**): mesenchyme (m), ventricular zone (vz), intermediate zone (iz), marginal zone (mz), floor plate (f), roof plate (r), lumen (l), dorsal ganglia (g), ventral horns (v), intermediate horns (i) and dorsal horns (d), ependymal layer (e). Insets show higher magnification of expression of serotonin receptors in motor neurons: sr1 (**j**), sr2 (**k**), sr3 (**l**), ×100. The intensity of sr1, sr2 and sr3 signals and differences in their overall expression pattern were measured (**n**), as well as differences in their corresponding signal in the motor neurons (**o**). Magnification ×40, scale bar 100 μm (**a**–**c**); magnification ×10, scale bar 400 μm (**d**–**g**,**j**–**m**); magnification ×20, scale bar 200 μm (**h**).

In the 5th and 6th developmental week, the moderate expression of serotonin receptor 1 (sr1) is observed in the developing human spinal cord, primarily in the ventricular and outer intermediate zones (Figure 2a). During the same developmental period, serotonin receptor 2 (sr2) shows a slightly lower expression than sr1, which extends throughout the whole width of the lateral spinal cord wall (Figure 2b). Serotonin receptor 3 (sr3) shows the weakest expression in the 5th and 6th developmental week, which extends throughout the lateral spinal cord wall (Table 1, Figure 2c,n).

In the 6th week and particularly in the 7th and 8th weeks, expression of sr1 further increases in the ventral part of intermediate zone (ventral horn with differentiating motor neurons), the floor plate and roof plate areas (Table 1, Figure 2d). During the same period, sr2 shows lower intensity than sr1 in the lateral wall, while intensity is stronger in floor and roof plate areas (Figure 1e). Sr3 shows the strongest expression in the 7–8-week spinal cord, particularly in the floor plate and roof plate areas, and in the ventral horns (Figure 2f).

In the 8th week, the transitional zone shows expression of sr1 around the perpendicular spinal cord lumens (ventricular zone), belonging to the cranial and caudal parts of the spinal cord, as well as in the outer border of the irregular intermediate zone (Figure 2g). Sr2 expression is weaker, but extends through the wall of the spinal cord, while sr3 shows weaker expression than sr1 and sr2 (Figure 2h,i).

In the 9th and 10th developmental week, sr1 shows mild expression in the marginal zone, moderate expression in the roof and floor plates and strong expression in ventral, intermediate and dorsal horns of the intermediate zone (Figure 2j). Compared to sr1, sr2 shows similar expression pattern, but expression in the ventral, intermediate and dorsal horns is less intense (Figure 2k), while expression of sr3 is less extensive in all analyzed parts of the spinal cord (Table 1, Figure 2l,o).

Insets of the higher magnification of the ventricular and intermediate zones (insets in Figure 2a–c), roof plate areas (Figure 2d–f) and the ventral horns (Figure 2j–l) reveal differences in the distributions and intensity of serotonin receptors. The differences around the motor neurons include the following: while sr1 forms accumulations of dense course granules around the motor neurons (inset of Figure 2j,o), sr2 forms less dense granulations (inset of Figure 2k,o). The expression signal of sr3 is mild and loosely distributed around the motor neurons (inset of Figure 2l,o).

The negative controls (omitting of primary antibodies) in all three srs show absence of signal, while the DAPI staining shows characteristic nuclear staining (Figure 2m).

### 2.4. Co-Expression of Serotonin Receptors with Neuronal Marker (pgp) and Glial Cell Marker (GFAP) (Double Immunofluorescence Staining)

**Figure 3:**
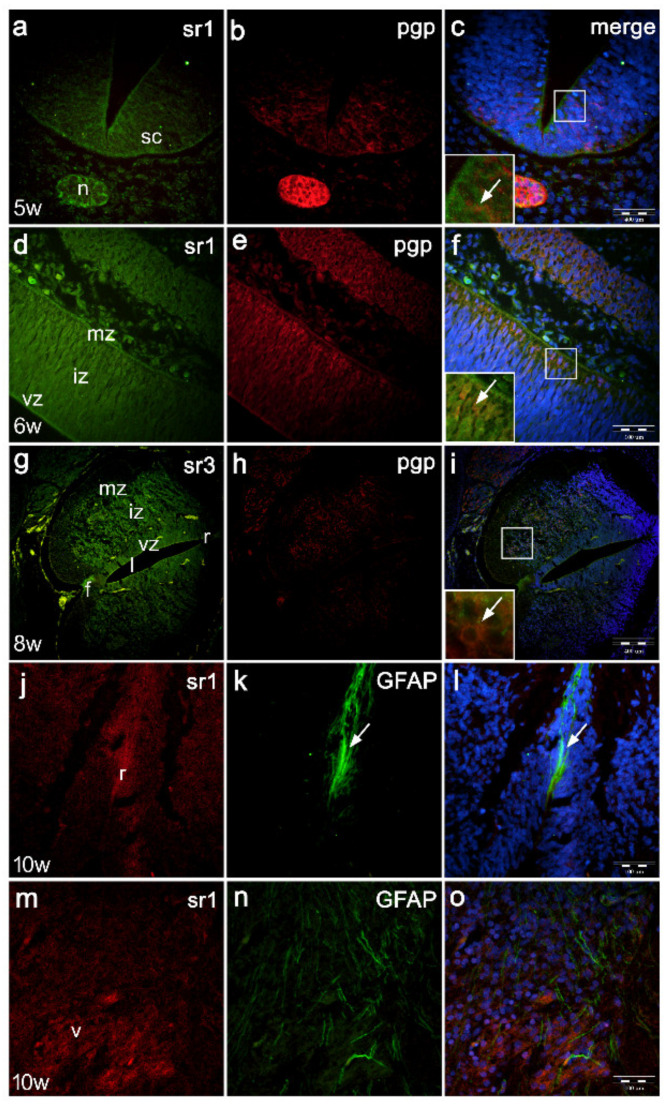
Double immunofluorescence staining of serotonin receptors with pan-neuronal marker (pgp) and glial fibrillary acidic protein (GFAP) in developing human spinal cord. Human spinal cord (sc) in the 5th developmental week (**a**–**c**), 6th week (**d**–**f**), 8th week (**g**–**i**), 10th week (**j**–**o**): ventricular zone (vz), intermediate zone (iz), marginal zone (mz), floor plate (f), roof plate (r), lumen (l), ventral horns (v), intermediate horns (i) and dorsal horns (d), notochord (n). In the 5th week co-expression of sr1 (**a**) and pgp (**b**) (inset in (**c**)) with DAPI nuclear stain (**c**) reveals co-expression of sr1/pgp in the intermediate zone neuroblasts (arrow) and in the notochord. Similar co-expression of sr1/pgp (inset in (**f**)) is observed in the outer part of the intermediate zone in the 6th week (**d**–**f**). In the 8th developmental week, sr3 (**g**) co-localizes with pgp (**h**) in the ventral horns (motor neurons) (inset in (**i**)) of the intermediate zone (**g**–**i**). Co-localization of sr1/GFAP is observed in the roof plate area (arrow) of the 10th week spinal cord (**j**–**l**), while it is absent in the ventral horn motor neurons (**m**–**o**). Magnification ×10, scale bar 400 μm (**a**–**i**); magnification ×40, scale bar 100 μm (**j**–**o**).

In the 5th developmental week, weak expression of sr1 is observed in the ventricular and marginal zones of the spinal cord. Sr1 is very weakly expressed in the intermediate zone and strongly in the nearby notochord (Figure 3a). Pan-neuronal marker (pgp) stains neuroblasts in the intermediate zone and very strongly stains the notochord (Figure 3b). Merging of the blue DAPI stain with sr1/pgp (Figure 3c) shows co-expression of Sr1 and pgp in the cells of intermediate zone containing neuroblasts (inset Figure 3c), as well as in the notochord cells (Figure 3c).

In the longitudinal section through the 6th week spinal cord, sr1 expression increases at the outer part of intermediate zone compared to earlier developmental stages (Figure 3d). Pgp strongly stains the most differentiated neurons at the outer part of intermediate zone (Figure 3e). Co-expression of DAPI nuclear stain with the two markers (Figure 3f) shows sr1/pgp co-localization in the outer part of intermediate zone (inset of Figure 3f).

In the 8th developmental week, sr3 expression is stronger in the lateral walls of the spinal cord than expression of sr1 and sr2. Roof and floor plate strongly express sr3 (Figure 3g). Expression of pgp is less extensive, particularly at lower magnifications, but characterizes the intermediate zone, which contains differentiating neurons (Figure 3h). Co-expression of blue DAPI nuclear stain with sr3/pgp (Figure 3i) reveals their co-localization in neuroblasts of the ventral horn in the marginal zone (inset of Figure 3i).

In the 10th developmental week, staining with antibodies for glial fibrillar acidic protein (GFAP) shows its expression in the lateral wall of the spinal cord, floor plate and roof plate areas. In the roof plate, both sr1 (Figure 3j) and GFAP (Figure 3k) display strong expression patterns. Overlapping of sr1/GFAP and DAPI nuclear stain shows their co-expression in the roof plate area (Figure 3l).

In the lateral wall of the 10th week spinal cord, ventral horns show strong expression of sr1 around the motor neurons (Figure 3m), while GFAP staining appears in the form of long radial glia, extending from ependymal cells towards the surface of the spinal cord (Figure 3n). When merging of DAPI nuclear stain with sr3/GFAP, the co-expression of the two markers is missing (Figure 3o).

### 2.5. Expression of Serotonin Receptors in the Parts of the Spinal Cord Caudal to Dysraphic Cervical Region in Human Fetus with Cervical Spina Bifida

The thoracic part of the spinal cord of human fetus with cervical spina bifida shows different types of histological morphological changes, particularly in the roof plate area (as described previously).

**Figure 4:**
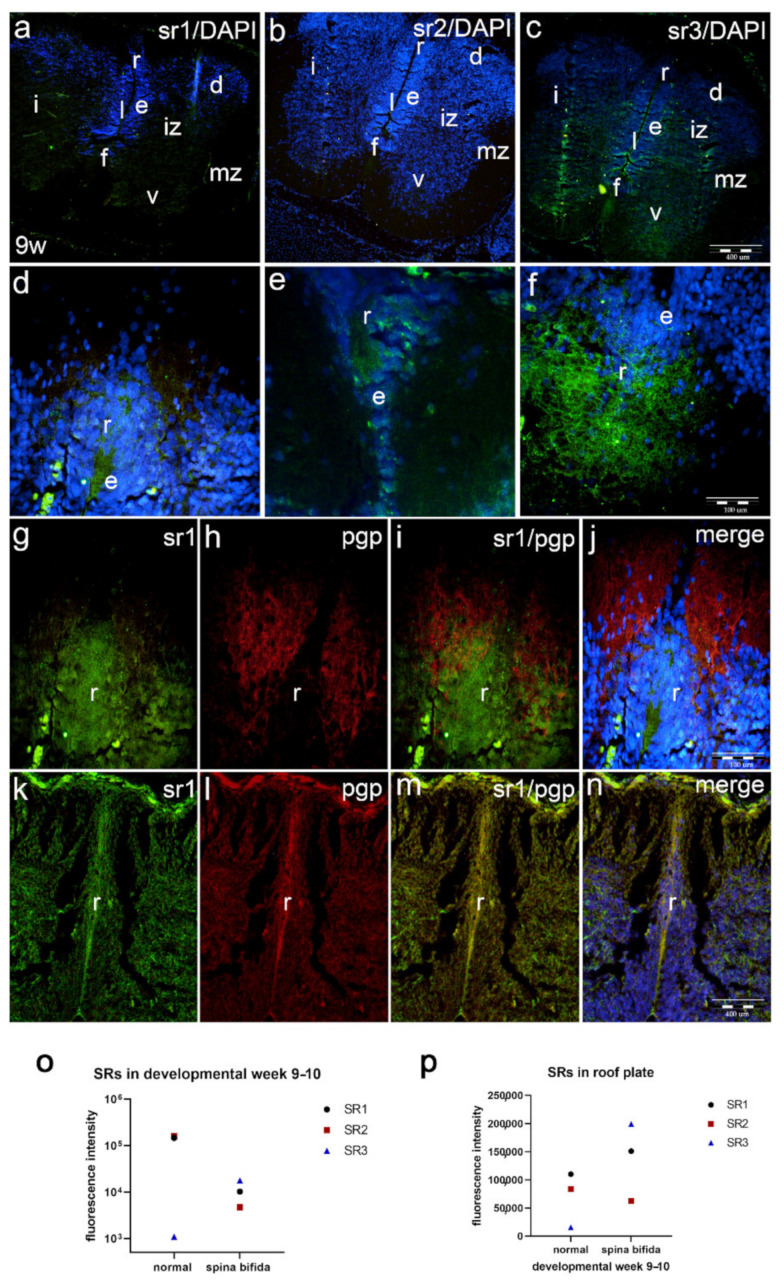
Immunofluorescence staining of the thoracic part of spinal cord in the fetus with cervical spina bifida with sr1, sr2 and sr3 and double immunofluorescence with pgp. Overall expression of sr1 (**a**), sr2 (**b**) and sr3 (**c**) shows differences in intensity of signal (**o**) when compared to normal spinal cord. Legend: spinal cord (sc), ependymal layer (e), intermediate zone (iz), marginal zone (mz), floor plate (f), roof plate (r), lumen (l), ventral horns (v), intermediate horns (i) and dorsal horns (d). The affected roof plate areas also show differences in intensity of sr1 (**d**), sr2 (**e**) and sr3 (**f**) signal (**p**). Double immunofluorescence staining of roof plate area in 9-week human fetus with cervical spina bifida (**g**–**j**) shows only narrow area of sr1/pgp co-localization. Control sample (**k**–**n**): very thin area of sr1/pgp co-localization; magnification ×10, scale bar 400 μm (**a**–**c**); magnification ×40 (**d**–**n**), scale bar 100 μm.

Immunohistochemical staining with sr1 and sr2 shows overall lower signal (Figure 4a,b) when compared to the normal spinal cord. The signal of sr3 is, on the other hand, stronger than in the normal spinal cord of the same developmental age (Figure 4c). We noticed clear differences in morphological appearance of the spinal cord in fetus with cervical spina bifida compared to the normal fetus (see Figure 2j–l) in cross section, particularly in the roof plate area.

Sr1 expression in the roof plate area shows stronger expression than in normal spinal cord. This is partly due to irregular appearance of the dorsal part of the spinal cord (Figure 4d). In contrast, sr2 expression is less extensive than sr1 in both normal fetus and fetus with spina bifida (Figure 4e). When stained with sr3, the roof plate in fetus with spina bifida shows stronger signal, both compared to normal fetus and compared to sr1 and sr2 signal in malformed fetus (Figure 4f).

Double immunofluorescence staining of sr1 (Figure 4g) and pgp (Figure 4h) mostly shows absence of co-expression of the two markers, except along the edge of the ventricular zone (ependymal cells) (Figure 4i); while sr1 predominantly co-localizes with cells corresponding to neuroepithelial (ependymal cells) in the hypertrophic roof plate area, pgp characterizes the marginal zone (axons of neurons) (Figure 4g–j).

Control samples of the roof plate area in the normal human spinal cord show lower signals of sr1 (Figure 4k) and pgp (Figure 4l) when compared to spina bifida, and partial co-localization of the two markers along the edge of ependymal cells (Figure 4m), as well as when co-localized with DAPI (Figure 4n). 

Statistical differences in intensity of sr1, sr2 and sr3 overall signal and signal in the roof plate areas are shown as Figure 4o,p.

### 2.6. Expression of Proliferation Marker Ki-67 and Apoptotic Marker Caspase-3 in the Roof Plate Area of Normal Fetus and Fetus with Spina Bifida (Immunofluorescence Staining)

**Figure 5:**
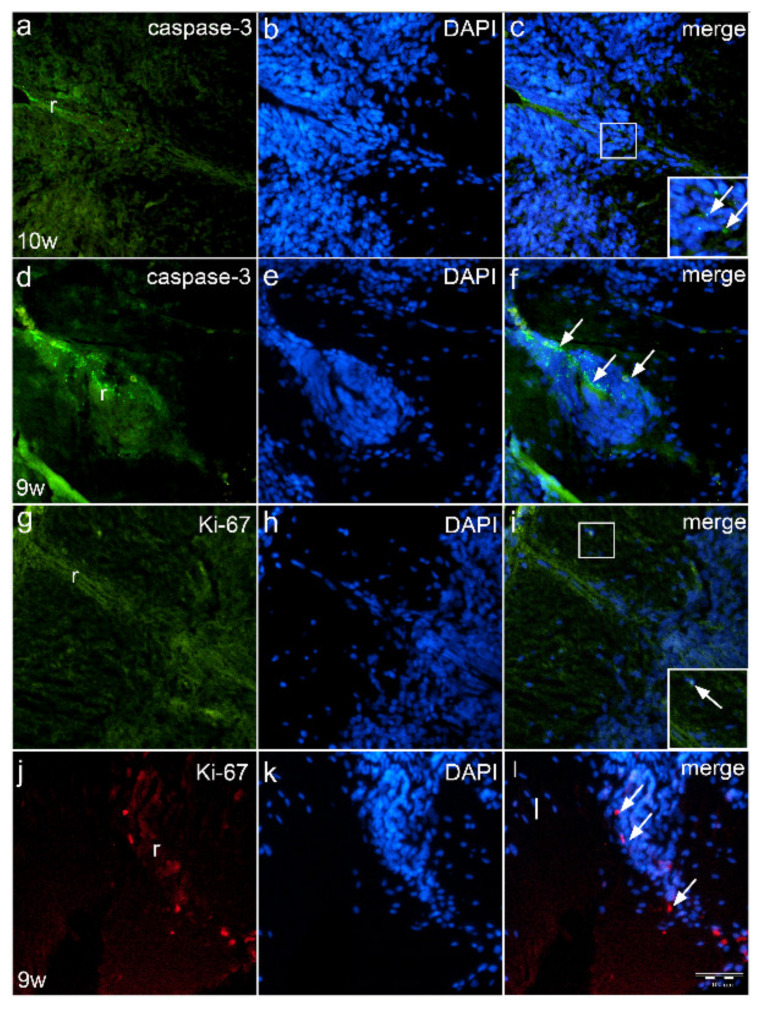
Immunofluorescence staining of roof plate area of normal human fetus and fetus with cervical spina bifida with apoptotic marker caspase-3 and proliferation marker Ki-67. Expression of caspase-3 apoptotic cells (arrows) in the roof plate (r) of normal 10th week embryo (**a**–**c**) has a different (regular) pattern when compared to the irregular distribution of caspase-3 positive cells (arrows) in the roof plate (r) of fetus with spina bifida (**d**–**f**). In normal fetus, Ki-67 positive cells are rarely seen (**g**–**h**), while in spina bifida, proliferating Ki-67 cells are irregularly organized (arrows) in the roof plate (r) (**j**–**l**). Magnification ×40, scale bar 100 μm (**a**–**i**).

In the 10th week of normal human fetus, caspase-3 positive apoptotic cells are observed in the dorsal part of the spinal cord, thus participating in the formation of the roof plate area (Figure 5a). Apoptotic cells appear predominantly in the form of apoptotic bodies aligned along the developing roof plate (inset of Figure 5c). Visualizing nuclei with DAPI staining shows the apoptotic cells to be positioned along the midline of the dorsal spinal cord (Figure 5b). Merging of caspase-3 and DAPI staining reveals the exact position of apoptotic cells in the midline of the dorsal spinal cord (Figure 5c).

In the 9th week, fetus with cervical spina bifida, thoracic spinal cord shows whirlpool-like appearance of the roof plate cells. Numerous apoptotic cells and apoptotic bodies are observed in the roof plate area (Figure 5d), which are found around the DAPI stained nuclei (Figure 5e) of the roof plate cells (Figure 5f).

In the 10th week normal fetus, Ki-67 positive cells (Figure 5g) are found only rarely near to the roof plate area. DAPI nuclear stain (Figure 5h) shows normal morphology of the roof plate, while Ki-67/DAPI co-localization (Figure 5i) shows nuclear co-expression of proliferating cells (inset of Figure 5i).

Ki-67 proliferating cells are also observed in the hypertrophic roof plate of the spinal cord in fetus with spina bifida (Figure 5j). DAPI stain shows morphologically different organization of nuclei in the roof plate of affected fetus (Figure 5k) compared to healthy fetus. Co-localization of Ki-67 positive cells with cell nuclei is observed (Figure 5l).

### 2.7. Expression of Serotonin Receptors in the Developing Dorsal Root Ganglia of Normal Fetuses and in Fetus with Cervical Spina Bifida (Immunofluorescence Staining)

**Figure 6:**
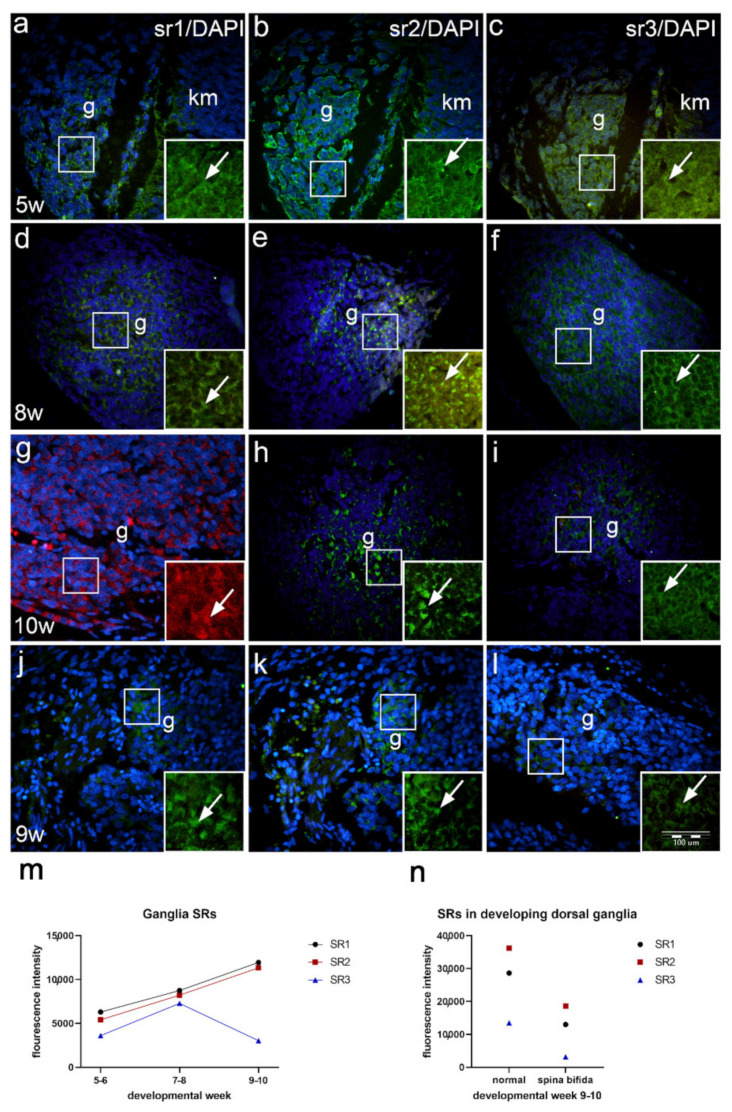
Immunohistochemical staining of sr1, sr2 and sr3 in developing human dorsal ganglia of normal fetus and in fetus with spina bifida. In the 5th developmental week, dorsal ganglia differ in the expression of sr1, sr2 and sr3 (**a**–**c**,**m**). In the 8th (**d**–**f**) and 10th developmental week (**g**–**i**) signal of sr1 and sr2 further intensifies (**m**), while sr3 decreases from the 8th week on (**m**). In the 9th week, fetus with spina bifida, sr1, sr2 and particularly sr3 show low intensity levels in ganglion cells (**j**–**l**,**n**). Legend: dorsal ganglion (g), expression of sr1, sr2 and sr3 (arrows in insets). Magnification ×40, scale bar 100 μm (**a**–**l**).

In the 5th week of development, the dorsal ganglia are situated on lateral sides of the developing spinal cord. Sr1 is moderately expressed in some ganglion cells (Figure 6a), while sr2 shows moderate expression (Figure 6b). Expression of the sr3 is weak compared to that of sr1 and sr2 (Table 2, Figure 6c,m).

During further development, dorsal ganglia enlarge in size. In the 8th developmental week, the majority of ganglion cells show moderate-to-strong sr1 expression (Figure 6d), while the signal of sr2 is stronger (Figure 6e) compared to sr1. Expression of sr3 is stronger that in earlier developmental stages, but its intensity is lower than the signal of sr1 and sr2 of the same stage (Figure 6f,m).

In the 10th developmental week, dorsal ganglia further enlarge, thus forming oval structures on both lateral sides of the spinal cord. The majority of ganglion cells show moderate-to strong expression of sr1 (Figure 6g), while expression of sr2 remains moderate (Figure 6h). On the other hand, expression of sr3 remains weak compared to earlier stages and to sr1 and sr2 signals in the 10th week (Figure 6i,m).

In the 9th week fetus with spina bifida, the dorsal ganglia do not appear oval (like normal dorsal ganglia) but loose and at places have a segmental appearance. Compared to normal ganglia of the same developmental stage, all serotonin receptors show lower signal intensity. Sr1 and sr2 are moderately expressed in some ganglion cells (Figure 6j,k). Sr3 signal is very weak and characterizes only a small population of ganglion cells (Table 2, Figure 6l,n).

### 2.8. Co-Expression of Serotonin Markers with pgp in Normal and Malformed 9–10-Week Human Fetus (Double Immunofluorescence Staining)

**Figure 7:**
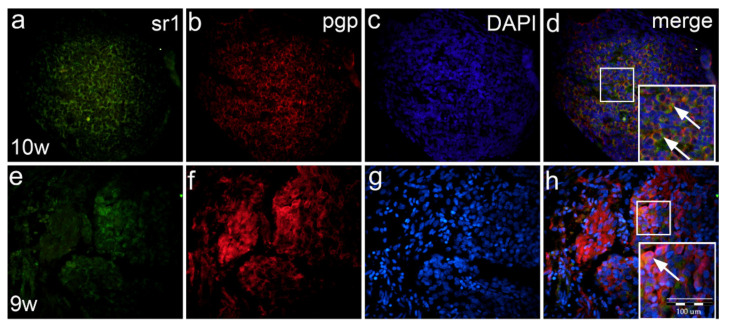
Double immunofluorescence staining of ganglia with serotonin markers and pgp in normal and malformed human fetus with spinal bifida. In the 10th week normal human dorsal ganglion (g), the majority of ganglion cells express sr1 (**a**) and pgp (**b**) markers. Co-localization of sr1/pgp with DAPI nuclear stain (**c**) shows their co-expression (arrow in insert) in most of the ganglion cells (**d**). In the 9th week fetus with spina bifida, sr1 is weakly expressed only in some ganglion cells (**e**). Co-localization with pgp marker (**f**) and DAPI (**g**) shows co-expression (arrow in the inset) of the sr1/pgp in some of dorsal ganglia cells (**h**). Magnification ×40, scale bar 100 μm (**a**–**h**).

In the 9th developmental week, sr1 is expressed moderately-to-strongly in nearly all dorsal ganglion cells (Figure 7a), while most of the ganglion neurons express the pgp marker as well (Figure 7b). Co-localization of Sr1/pgp with DAPI nuclear stain (Figure 7c) reveals that the majority of neurons co-express both sr1 and pgp in the cytoplasm of the same cells (Figure 7d inset).

In 9th week human fetus with spina bifida, sr1 is less expressed (Figure 7e) when compared to normal ganglia. Pgp marker stains ganglion neurons and nerve fibers (Figure 7f), while DAPI stains nuclei of ganglion cells and Schwann cells (Figure 7g). Following merging, co-expression of sr1 and pgp is observed in ganglion cells, but not in the entire population (Figure 7h inset).

## 3. Discussion

### 3.1. Development of Serotonergic Receptors during Early Development of the Human Spinal Cord

Our study has shown that already in the earliest stages of human development, sr1 expression characterized the ventricular and outer intermediate zones of the developing spinal cord. When compared to sr1, sr2 and sr3 showed weaker expression, which was uniformly distributed throughout the lateral wall of the spinal cord. Such findings indicate early influence of the sr1 on the proliferation of neuroepithelial cells in the ventricular zone and parallel stimulation of neuroblast differentiation in the intermediate zone. During further development, sr expression increased in the ventral horns populated by the motor neurons and subsequently, with lower intensity, in the intermediate and dorsal horns, containing autonomic and sensory neurons, respectively. The co-localization of serotonin receptors with neuronal markers was observed in all three horns of the intermediate zone, thus indicating importance of serotonin in the differentiation of different types of neurons (motor neurons, autonomic and sensory neurons). However, at later developmental stages, expression of sr2 and sr3 increased primarily in the floor and roof plates, where they co-localized with glial markers, thus suggesting influence of those two receptors on the morphogenesis of ventral and dorsal midline spinal cord areas. In the 10th developmental week, overall expression of sr1 and sr2 in the spinal cord increased, while that of sr3 decreased. Our study indicated differences in the localization, expression intensity and developmental role of the three investigated serotonin receptors during the normal development of the human spinal cord.

Similar to our study, previous studies on experimental animals also showed that serotonin plays the role of a growth factor in the immature mammalian brain—directing both proliferation and maturation of neurons. Moreover, the turnover rate of serotonin was higher in the immature neural tissue than at any other time in life. In addition, other factors were shown to influence serotonergic neuron development, such as dopamine, which displayed an inhibitory effect, while substance P and ACTH showed stimulatory effects [13]. In the human embryos, neurons positive to AChE, substance P and encephalins were found in all regions of the intermediate zone of the spinal cord, thus pointing to possible modulation of cholinergic autonomic activities by neuropeptides [26]. Analysis of the 5-HT1a receptor during animal development showed that it appeared only at specific periods of development in high amounts and then decreased with aging, while the 5-HT2 receptor was not detectable during development at all, which is opposite to our findings in human development. Investigations on animal models showed that activation of 5-HT3 receptors displayed inhibitory effects to the development of serotonin neurons [13]. Investigation of the late fetal period in humans (21–32-weeks) revealed that 5-HTA2 and 5-HT2C receptors increased in the developing thalamus, while neither neuronal cells nor fibers displayed any immunoreactivity for 5-HT3 or 5-HT6 [27]. In contrast to those findings, our investigation of embryonic and early fetal stages of human development showed the importance of 5-HT3 in the floor and roof plate formation during the early morphogenesis of the spinal cord. Concerning rare studies on humans, the early expression of various transmitters and peptides has been documented in the human CNS during embryonic and fetal developmental stages, and those also included serotonin and its receptors. Many of these transmitters and peptides, including serotonin, declined during normal and abnormal aging as a consequence of cell death or otherwise [12]. In accordance with our study, it was shown that the serotonergic medullary neurons sent bulbospinal axons to all laminae of the spinal cord and innervated medullary sensory, motor, autonomic and respiratory nuclei. In the spinal cord, the serotonergic receptors were primarily located on axon terminals. Benarroch et al. reported that 5-HT1 receptors have an inhibitory effect and reduce neuronal firing, while 5-HT2 receptors primarily exerted an excitatory effect. The 5-HT3 receptors were suggested to be nonselective cation channels that elicited fast depolarization. Activation of serotonergic receptors was shown to induce a general increase in the excitability of motor neurons through the modulation of several classes of ion channels [14].

### 3.2. Expression of Serotonin Receptors in Malformed Human Fetus with Cervical Spina Bifida

Analysis of human fetus with cervical spina bifida revealed different types of morphological changes along the thoracic part of the spinal cord caudal to its dysraphic cervical part: from hypertrophy of the marginal layer, to rolling up of one lateral spinal cord walls over another. In all cases the roof plate area had different shapes when compared to its morphology in normal spinal cord. In addition to morphological changes, the spinal cord of malformed fetus showed lower overall intensity of sr1 and sr2 signals, and higher intensity of the sr3 signal in comparison to normal spinal cord of the same developmental stage. Irregularities in the morphology and appearance of the serotonin receptor signal was observed in the roof plate, where the sr3 signal showed stronger intensity than sr1 and sr2 signals. In addition, we detected a narrow area of co-localization of serotonin receptors with pan-neuronal markers, presence of proliferating cells as well as increased accumulation of apoptotic cells in the morphologically changed roof plate of malformed fetus. Based on these findings, we suggest that the process of proliferation could be associated with efforts of closing the dorsal part of the spinal cord, while apoptosis could enable later re-modelling of the affected roof plate area.

In the animal models, SCI was shown to induce variable alterations in 5-HT projections, depending on the type of injury, lesion severity and animal used. In addition, similar to our findings, in the rat spinal cord immediately caudal to the lesion, 5-HT immunoreactivity was significantly reduced compared to normal, while it was less affected further caudally [28,29]. Following SCI, 5-HT axons caudal to the lesion site usually degenerated, while rostral to the lesion they sprouted, irrespectively of the severity of the injury. It was shown that activation of the specific receptors may increase 5-HT neurotransmission and promote functional recovery after SCI [20,21]. On the other hand, 5-HT axon sprouting following SCI could be inhibited by the presence of myelin-derived inhibitor proteins, which binds to the Nogo-66 receptor (NgR) on 5-HT neurons and may prevent their sprouting and regeneration of the spinal cord. In the malformed fetus described in this study, the dorsal part of the spinal cord was heavily malformed and we noticed axon sprouting caudal to the lesion, probably in order to close the opened roof plate area. A similar process was observed in the case of dorsal hemisection in mice [30] or moderate contusion in rats [31] (see Supplementary Figure S1), when pan-neuronal overexpression of an endogenous NgR antagonist was associated with 5-HT axon sprouting caudal to the lesion site [32]. In addition, genetic deletion of NgR in mice enabled regeneration of 5-HT axons across the lesion site following complete section of the spinal cord [33].

The described characteristics of the human pathologically changed spina bifida only partly corresponded to changes described during the scarring process in adult mice following dorsal spinal cord hemisection [34]. Namely, in the mice scar area, the reactive astrocytes at the scar periphery produced molecules that increased the sprouting process of serotonergic axons. In our study, we noticed that GFAP expression in the radial glia and roof plate area, as well as stronger pgp expression in the roof area of abnormal fetus with spina bifida were associated with increased sr expression, particularly sr3. During development of the roof plate in the human spinal cord, the bipotential progenitor cells in the ventricular zone (neuroepithelium) may give rise to both neuronal (expressing pgp) and glial progenitors (expressing GFAP), which during further development can differentiate into astrocytes, oligodendrocytes and radial glia [35]. However, at this early stages of human spinal cord development described in this study, immature astrocytes were still in the stage of their progenitor cells—radial glia.

Spasticity, which has been observed after the SCI, may reflect the interaction of several mechanisms, including injury of serotonergic axons that innervate the motoneurons. Increased motoneuron excitability may be caused by the 5-HT2A receptor–mediated activation of a persistent Ca^2+^ current, followed by later compensatory upregulation of 5-HT2B and 5-HT2C receptors. Following the SCI, reduction of 5-HT1 receptor activity contributed to persistent depolarization of motor neurons during muscle spasms [14]. Again, 5-HT-mediated modulation of motor neuron excitability was believed to depend on the level of the neurotransmitter released and on the specific location of 5-HT receptors in the spinal cord [23]. Experimental transplantation of B1, B2 and B3 raphe nuclei caused re-innervation of their specific targets [36], as the transplant-derived 5-HT neurons survived and integrated within the injured host spinal cord in rats [37,38].

### 3.3. Expression of Serotonin Receptors in Developing Spinal Ganglia and Ganglia of Fetus with Spina Bifida

During the analyzed phases of human development, spinal ganglia enlarged while their expression of serotonin receptors displayed a specific temporal pattern. Thus, sr1 showed the strongest expression in dorsal ganglia, while expression of both sr1 and sr2 increased from moderate to strong. The overall expression of sr3 was weaker than expression of sr1 and sr2 but had a peak of intensity during the 7th and 8th developmental week. In our study, 5-HTreceptors mostly co-localized with the pan-neuronal marker in the dorsal ganglion neurons. In the 9th-week fetus with spina bifida, we observed morphological abnormalities and reduced expression of all three serotonin receptors when compared to normal fetuses of the same age. Previous studies on serotonin expression in dorsal ganglia of different animals resulted in controversial data. Thus, the number of 5-HT-expressing neurons in dorsal ganglia varied between studies, depending on the species used and dorsal ganglion level. the 5-HT-expressing neurons were recognized as a key subpopulation during dorsal ganglion development [39], particularly 5-HT5A and 5-HT5B, which were observed only during the embryonic period. Other different subtypes of 5-HT receptors present in the adult dorsal ganglia were shown to have distinct roles in sensory neurotransmission [40], as the majority of small and large size neurons were 5-HT positive in the dorsal root ganglion [41].

## 4. Materials and Methods

### 4.1. Human Samples

A total of 15 embryonic and fetal spinal cord tissues were taken from the conceptuses of the estimated age between the 5th and 10th developmental week. The used human material belongs to the archival collection of histological sections stored in the Department of Anatomy, Histology and Embryology, School of Medicine, University of Split. Conceptuses were collected after spontaneous abortions or tubal pregnancies and carefully examined afterwards. Only normal fetuses without any abnormal morphological signs or macerations were used and processed in this study. The postovulatory age of the conceptuses was determined with the menstrual cycle data and crown-lump length and were correlated with the Carnegie stages [42]. We also included tissue of a 9-week fetus with cervical spina bifida [5]. Tissue was collected from the Department of Gynecology and Obstetrics and the Department of Pathology of the University Hospital Center Split. Tissue collecting and processing was performed in accordance with the Helsinki Declaration and the permission of the Ethical and Drug Committee of the University Hospital of Split (class: 003-08/16-03/0001, registry number: 2181-198-03-04-16-0024, 20 May 2016).

### 4.2. Hematoxylin and Eosin

Following the fixation in 4% paraformaldehyde in phosphate buffer saline (PBS), tissue was dehydrated in graded ethanol solutions. Tissue was then embedded in paraffin blocks, cut serially as 5–7 µm-thick sections and mounted on glass slides. H&E (hematoxylin and eosin) staining was performed in order to describe stages in normal spinal cord development and anomalous changes of the thoracic and lumbar parts of the fetus with cervical spina bifida.

### 4.3. Immunohistochemistry and Immunofluorescence Staining

Tissue underwent deparaffinization in xylol and rehydration in graded ethanol as previously described [43]. After heating in a sodium citrate buffer for 20 min at 95 °C, a cooling period was required for tissues to settle to room temperature. To prevent non-specific staining, after washing the tissue with PBS, blocking buffer (Protein Block ab64226, Abcam, Cambridge, UK) was administered on the tissue covered area of the slides for 30 min. After the next rinsing in PBS, samples were incubated overnight with the primary antibodies in a humid chamber (Table 3). Application of secondary antibodies succeeded rinsing the tissue with PBS, with incubation in a humid chamber for one hour (Table 3). Finally, tissues were rinsed in PBS one more time, and nuclei were counterstained with DAPI (4′,6-diamidino-2-phenylindole) for 2 min, washed with PBS and then cover-slipped (Immuno-Mount, Thermo Shandon, Pittsburgh, PA, USA). For the control of the specificity, primary antibodies were omitted from the protocol, and no staining appeared. The microscope used for observation and imaging was an Olympus fluorescence microscope (BX61; Tokyo, Japan) with a digital camera (DP71) [44].

### 4.4. Semi-Quantification

Intensity of staining with antibodies chosen in our study was classified into four categories: (+/−) indicating weak expression, (+) as mild expression; (++) as moderate expression; and (+++) as strong expression (Table 1 and Table 2). Three experienced separate researchers semi-quantitatively analyzed the staining intensity using image analysis software ImageJ (National Institutes of Health, Bethesda, MD, USA) [44].

### 4.5. Statistics and Microphotograph Quantification

Figures were prepared for analysis firstly by acquiring fluorescence intensity histograms for green fluorescence channel in ImageJ software (NIH, Bethesda, MD, USA). Background threshold as well as threshold for artifacts were set by three experienced histologists. Expression of different proteins was quantified as the area under the curve (AUC) of florescence intensity histograms (see Supplementary Figures S2–S5). AUC is a product of the number of pixels covered by signals and their intensity; therefore, in one “measure”, it contains both important features of expression, i.e., special arrangement (area) and intensity of signal. AUCs and their interval estimates were calculated by using AUC analysis routine in GraphPad Prism 9.1 software (Graph Pad, La Jolla, CA, USA). Expression of serotonin during embryonic and fetal development was analyzed by finding peaks or nadirs and trends in developmental weeks using the single sample or paired *t*-test or by linear regression. We analyzed 2 to 4 sections per embryo, and we had 2 embryos for each timepoint. Statistical significance, effect sizes, measures of goodness of fit as well as respective 95%CI were calculated in GraphPad Prism 9.1 software (Graph Pad, La Jolla, CA, USA). Level of significance was set at *p*  =  0.05 [44].

## 5. Conclusions

In early human development, soon after accomplishment of the neurulation process, the mild or moderate expression of all three serotonin receptors (sr1, sr2 and sr3) was observed in the developing neuroblasts of the spinal cord and ganglia. During their further maturation, sr expression increased with different intensity in the motor neurons of ventral horns, followed by their gradual appearance in autonomic and sensory neurons of intermediate and dorsal horns. The observed expression pattern indicated the importance of sr1 primarily in neuronal differentiation, while sr2 and sr3 had a role in the control of the floor plate and roof plate morphogenesis. In the developing ganglia, sr1 and sr2 seemed to have a role in neuronal maturation throughout the investigated period, while the increased expression of sr3 was observed in ganglia during a limited period of time. In the abnormal fetus with spina bifida, in addition to morphological abnormalities of spinal cord layers and roof plate area, the overall sr signaling was weaker both in the spinal cord and ganglia when compared to normal, while roof plate areas showed increased sr3 expression. The roof plate area was additionally characterized by the presence of apoptotic and proliferating cells, probably serving in closure and re-modeling of the affected dorsal spinal cord. Our study revealed human species-specific expression pattern of serotonin receptors in the developing human spinal cord and ganglia, and their disturbed expression associated with an abnormal neurulation process in human fetus with spina bifida.

## Figures and Tables

**Table 1:** Expression of serotonin receptors in developing human spinal cord by semi-quantification.

Developmental Weeks	Spinal Cord Parts	sr1	sr2	sr3
5	vz	+/++	+/++	+/−
	iz	+/++	+/++	+/−
	mz	+/−	+/++	+/−
6	vz	+/++	+/++	+
	iz	++	+/++	+
	mz	+	+	+/−
	rp	+/++	++	+/++
	fp	+/++	++	+/++
7 and 8	vz	+/++	+/++	++/+++
	iz–vh	++	+	++
	iz–ih	+	+	+
	iz–dh	+	+	+
	mz	+	+	+
	rp	++	++/+++	+++
	fp	++	++/+++	+++
9 and 10	el	++	++	++
	iz–vh	+++	++/+++	++
	iz–ih	+++	++/+++	++
	iz–dh	+++	++	++
	mz	+	+	+
	rp	++	++	++
	fp	++	++	++

Ventricular zone (vz), intermediate zone (iz), marginal zone (mz), roof plate (rp), floor plate (fp), ventral horn (vh), intermediate horn (ih), dorsal horn (dh), ependymal layer (el). (+/−) weak expression; (+) mild expression; (++) moderate expression; (+++) strong expression.

**Table 2:** Expression of serotonin receptors in developing human spinal ganglia by semi-quantification.

Developmental Weeks	sr1	sr2	sr3
5–6	+	++	+/−
7–8	++	++/+++	+/++
9–10	++	++/+++	++
9 (cervical spina bifida)	+/++	++	+

Ventricular zone (vz), intermediate zone (iz), marginal zone (mz), roof plate (rp), floor plate (fp), ventral horn (vh), intermediate horn (ih), dorsal horn (dh), ependymal layer (el). (−) weak expression; (+) mild expression; (++) moderate expression; (+++) strong expression.

**Table 3:** Primary and secondary antibodies.

	Antibody	Code No.	Host	Dilution	Source
Primary	Anti-5HT1A Receptor antibody	ab227165	Rabbit	1:100	Abcam
	SR-2A (A-4)	sc-166775	Mouse	1:100	Santa Cruz Biotechnology Inc.
	5-HT3A receptor antibody	GTX54151	Rabbit	1:100	GeneTex
	PGP9.5 Monoclonal Antibody (BH7)	480012	Mouse	1:500	Invitrogen
	anti-GFAP antibody	ab53554	Goat	1:100	Abcam
	anti-GFAP (2E1)	sc-33673	Mouse	1:50	Santa Cruz Biotechnology Inc.
	anti-Ki-67	M7240	Mouse	1:100	DAKO
	Caspase-3 antibody	AF835	Rabbit	1:1500	R&D System, Inc.
Secondary	Alexa Fluor^®^488 AffiniPure Anti-Mouse lgG (H+L)	715-545-150	Donkey	1:400	Jackson Immuno Research Laboratories
	Alexa Fluor^®^488 AffiniPure Anti-Goat lgG (H+L)	705-545-003	Donkey	1:400	Jackson Immuno Research Laboratories
	Alexa Fluor^®^488 AffiniPure Anti-Rabbit lgG (H+L)	711-545-152	Donkey	1:400	Jackson Immuno Research Laboratories
	Rhodamine Red™-X (RRX) AffiniPure Anti-Goat IgG (H+L)	705-295-003	Donkey	1:400	Jackson Immuno Research Laboratories
	Rhodamine Red™-X (RRX) AffiniPure Anti-Mouse IgG (H+L)	715-295-151	Donkey	1:400	Jackson Immuno Research Laboratories

## Data Availability

The data presented in this study are available on request from the corresponding author. The data are not publicly available due to ethical restrictions.

## Supplementary Materials

The following are available online at https://www.mdpi.com/article/10.3390/ijms22147320/s1, Figure S1: Schematic drawing showing differences between neuronal sprouting in the spinal cord following dorsal hemisection in rodents and spina bifida in humans, Figure S2: Representative histograms of Fluorescence Intensity in the spinal cord, Figure S3: Representative histograms of Fluorescence Intensity in the roof plate, Figure S4: Representative histograms of Fluorescence Intensity in the dorsal root ganglia, Figure S5: Representative histograms of Fluorescence Intensity in the motor neurons.
