# Supplementary material for: Expression Pattern of 5-HT (Serotonin) Receptors during Normal Development of the Human Spinal Cord and Ganglia and in Fetus with Cervical Spina Bifida
Source: Int J Mol Sci. 2021 Jul 7;22(14):7320. doi: 10.3390/ijms22147320 (PMC8304340; doi:10.3390/ijms22147320)
Supplement: Supplementary file 1 [file ijms-22-07320-s001.zip › ijms-1222258-supplementary.pdf]

Supplementary Material

Expression Pattern of 5-HT (Serotonin) Receptors during Normal Development of the Human Spinal Cord and Ganglia and in Fetus with Cervical Spina Bifida

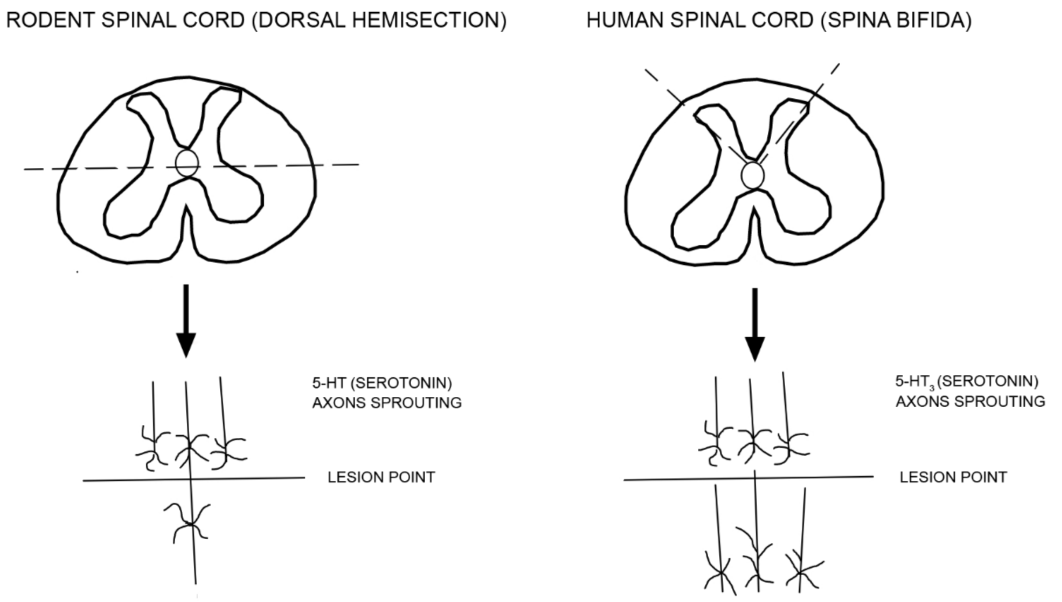

Figure S1. Schematic drawing showing differences between neuronal sprouting in the spinal cord following dorsal hemisection in rodents and spina bifida in humans.

Representative histograms of Fluorescence Intensity

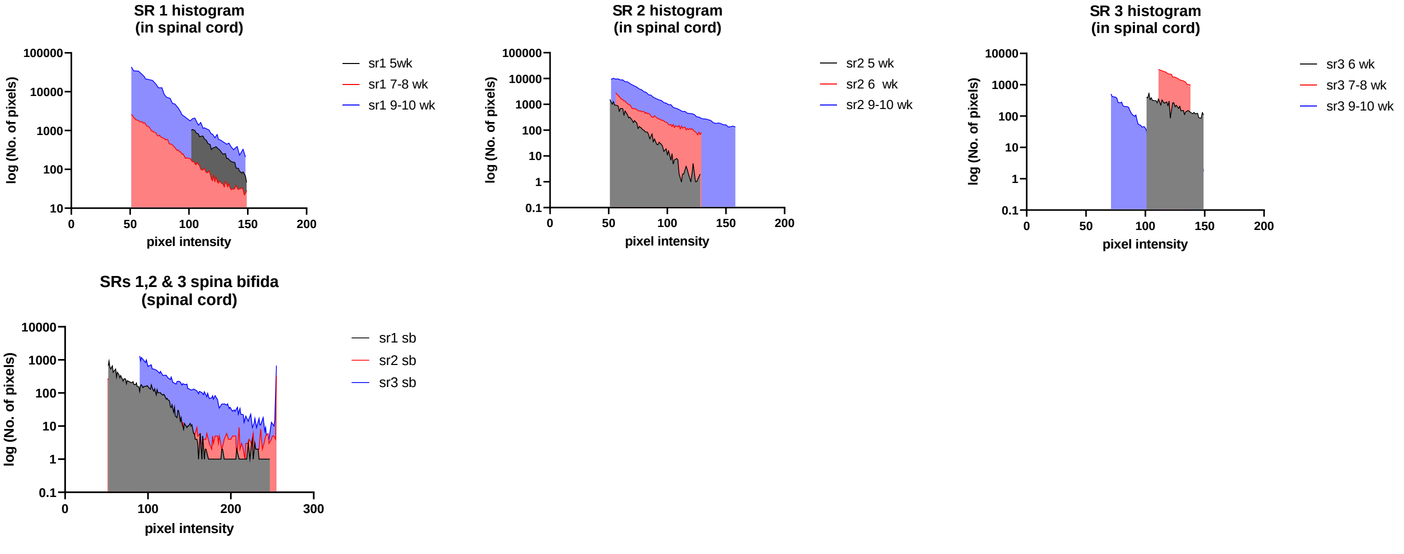

Figure S2. Representative histograms of Fluorescence Intensity in the spinal cord.

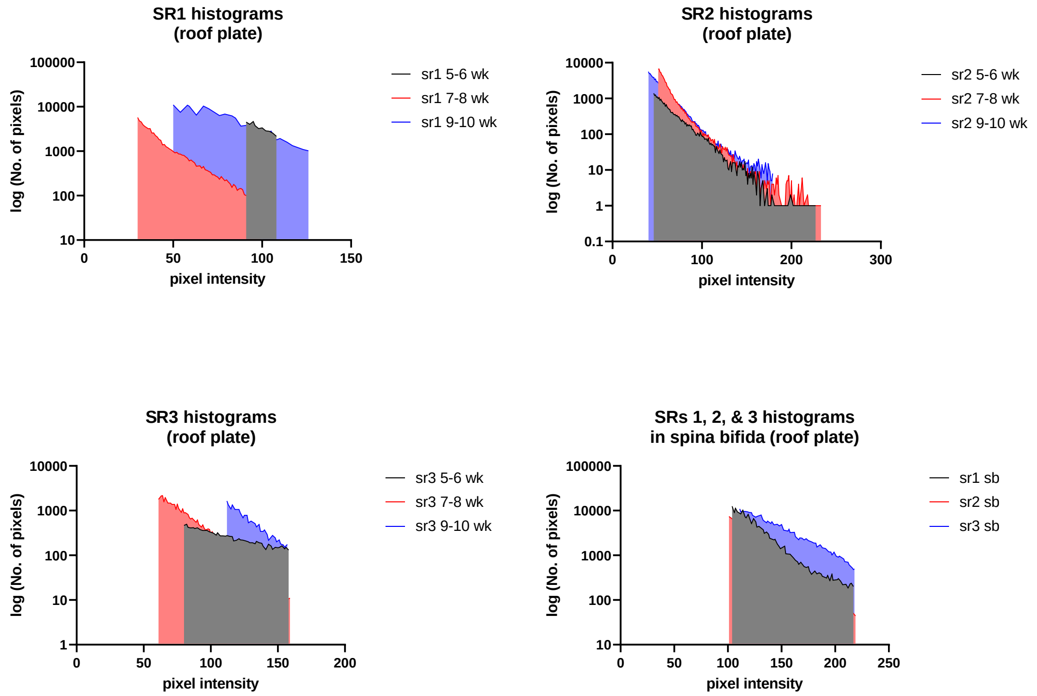

Figure S3. Representative histograms of Fluorescence Intensity in the roof plate.

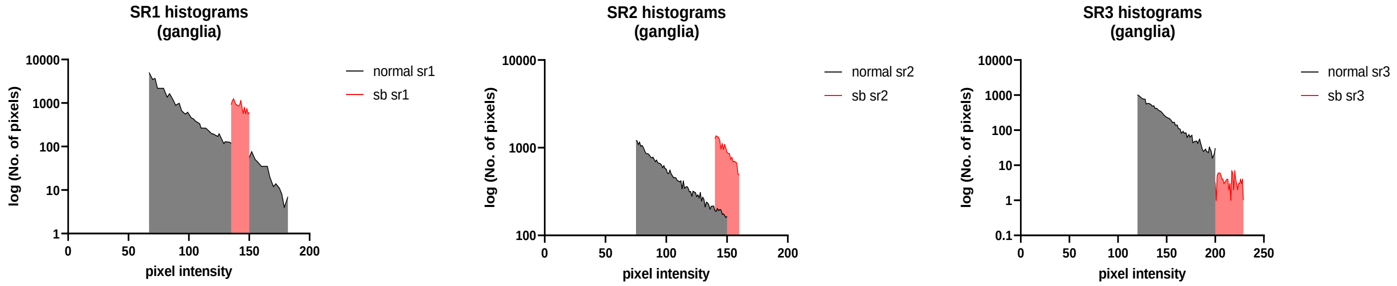

Figure S4. Representative histograms of Fluorescence Intensity in the dorsal root ganglia.

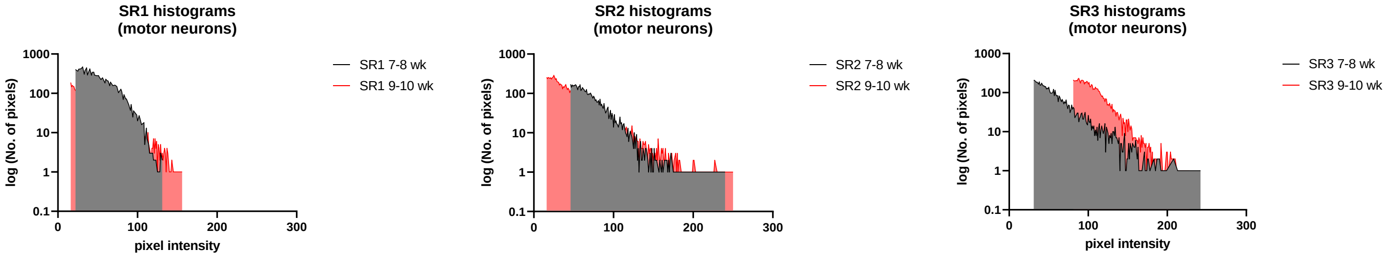

Figure S5. Representative histograms of Fluorescence Intensity in the motor neurons.
